# Molecular regulation and physiological functions of a novel *FaHsfA2c* cloned from tall fescue conferring plant tolerance to heat stress

**DOI:** 10.1111/pbi.12609

**Published:** 2016-09-23

**Authors:** Xiuyun Wang, Wanlu Huang, Jun Liu, Zhimin Yang, Bingru Huang

**Affiliations:** ^1^ College of Agro‐Grassland ScienceNanjing Agricultural UniversityNanjingChina; ^2^ Department of Plant Biology and Pathology, Rutgersthe State University of New JerseyNew BrunswickNJUSA

**Keywords:** tall fescue, HsfA2c, HSPs, heat stress, physiological functions, thermotolerance

## Abstract

Heat stress transcription factors (*HSFs*) compose a large gene family, and different members play differential roles in regulating plant responses to abiotic stress. The objectives of this study were to identify and characterize an A2‐type HSF,* FaHsfA2c,* in a cool‐season perennial grass tall fescue (*Festuca arundinacea* Schreb.) for its association with heat tolerance and to determine the underlying physiological functions and regulatory mechanisms of *FaHsfA2c* imparting plant tolerance to heat stress. *FaHsfA2c* was localized in nucleus and exhibited a rapid transcriptional increase in leaves and roots during early phase of heat stress. Ectopic expression of *FaHsfA2c* improved basal and acquired thermotolerance in wild‐type *Arabidopsis* and also restored heat‐sensitive deficiency of *hsfa2* mutant. Overexpression of *FaHsfA2c* in tall fescue enhanced plant tolerance to heat by triggering transcriptional regulation of heat‐protective gene expression, improving photosynthetic capacity and maintaining plant growth under heat stress. Our results indicated that FaHsfA2c acted as a positive regulator conferring thermotolerance improvement in *Arabidopsis* and tall fescue, and it could be potentially used as a candidate gene for genetic modification and molecular breeding to develop heat‐tolerant cool‐season grass species.

## Introduction

Supraoptimal temperature is a primary stress for cool‐season plant species during summer months (Wahid *et al*., 2007), which influences an array of processes including physiological growth, yield and quality of crop (Prasad *et al*., 2008). Heat stress response (HSR) in plants is highly complicated, involving multiple signal pathways, regulatory networks, and physiological and biochemical processes (Kotak *et al*., 2007; Zhou and Abaraha, 2007). Early perception and transduction of heat stress signal in plants are mainly mediated by heat stress transcription factors (HSFs), which are the terminal components of the signal transduction chain and trigger transcription cascades of downstream heat‐responsive genes (Mittler *et al*., 2012). HSFs compose a large gene family of 26 members in rice (*Oryza sativa*) (Mittal *et al*., 2009) and 21 members in *Arabidopsis thaliana* (Nover *et al*., 2001). Different members of HSFs in plants are involved in different functions controlling multiple aspects of HSR that confer heat tolerance (Scharf *et al*., 2012).

According to the structural features of oligomerization domains (OD), all the HSF family members can be classified into three major groups: HsfA, B and C (Nover *et al*., 2001). Among the whole HSF family, HsfA2s are the most heat‐responsive and strongly expressed members in HSR and play important roles in basal and acquired thermal tolerance (Scharf *et al*., 2012). Expression of *HsfA2* is strictly heat stress dependent in *Arabidopsis,* which regulates a subset of downstream heat‐response genes, including ascorbate peroxidase (*Apx2*) and a series of heat‐shock proteins (*HSPs*) (Schramm *et al*., 2006). *HsfA2‐*knockout plants showed a significant heat‐sensitive phenotype from *Arabidopsis* T‐DNA insertion mutants of 48 heat‐induced genes, and the heat‐sensitive deficiency was rescued by introducing a wild‐type copy of *HsfA2* (Charng *et al*., 2007). In addition to its role in heat tolerance, HsfA2s play important roles in anoxia tolerance (Banti *et al*., 2010), salt or osmotic stress tolerance and in the regulation of cell proliferation (Ogawa *et al*., 2007). Up to date, investigation on HsfA2s is mainly focused on *Arabidopsis* and tomato, which only possess one HsfA2, whereas monocot plants usually have up to five members (HsfA2a to e) and each HsfA2 member may play specific or unique roles in HSR (von Koskull‐Doering *et al*., 2007). Five members of OsHsfA2s were identified, which all responded to heat stress but OsHsfA2c was considered to play a major role in rice responses to heat stress (Yokotani *et al*., 2008). HvHsfA2c was also found in barley (*Hordeum vulgare*) (Mangelsen, 2010), but the functions of HsfA2c in relation to basal or acquired thermotolerance have not been well documented.

Although increasing number of HSF members have been found along with genome sequencing of many grass species, such as rice, barley and wheat (*Triticum aestivum*), little is known about physiological functions and regulatory molecular factors of specific member of HsfA2s involved in grass tolerance to heat stress, particularly perennial grass species, which experience repeated heat stress during the summer. Many stress‐responsive genes, such as mannitol 1‐phosphate dehydrogenase (*mt1D*) and dehydration‐response element binding protein (*DREB*), have been genetically transformed into forage and turfgrass to improve drought, freezing and salt tolerance, but there were few studies on heat tolerance (Wang and Ge, 2006). The objectives of this study were to identify and characterize an A2‐type HSF, FaHsfA2c, in a perennial grass tall fescue for its association with heat tolerance, and to determine the underlying physiological functions and regulatory mechanisms of FaHsfA2c imparting plant tolerance to heat stress. Identification and characterization of *FaHsfA2c* were performed by cloning and examining its expression patterns in response to heat stress, as well as subcellular localization. The physiological functions of FaHsfA2c conferring heat tolerance were addressed by overexpressing *FaHsfA2c* in wild‐type *Arabidopsis and hsfa2* insertion mutants, as well as tall fescue through measurements of several major physiological and biochemical parameters associated with heat tolerance.

## Results

### *FaHsfA2c* encoding an A2‐type HSF localized in nucleus

**Figure 1:**
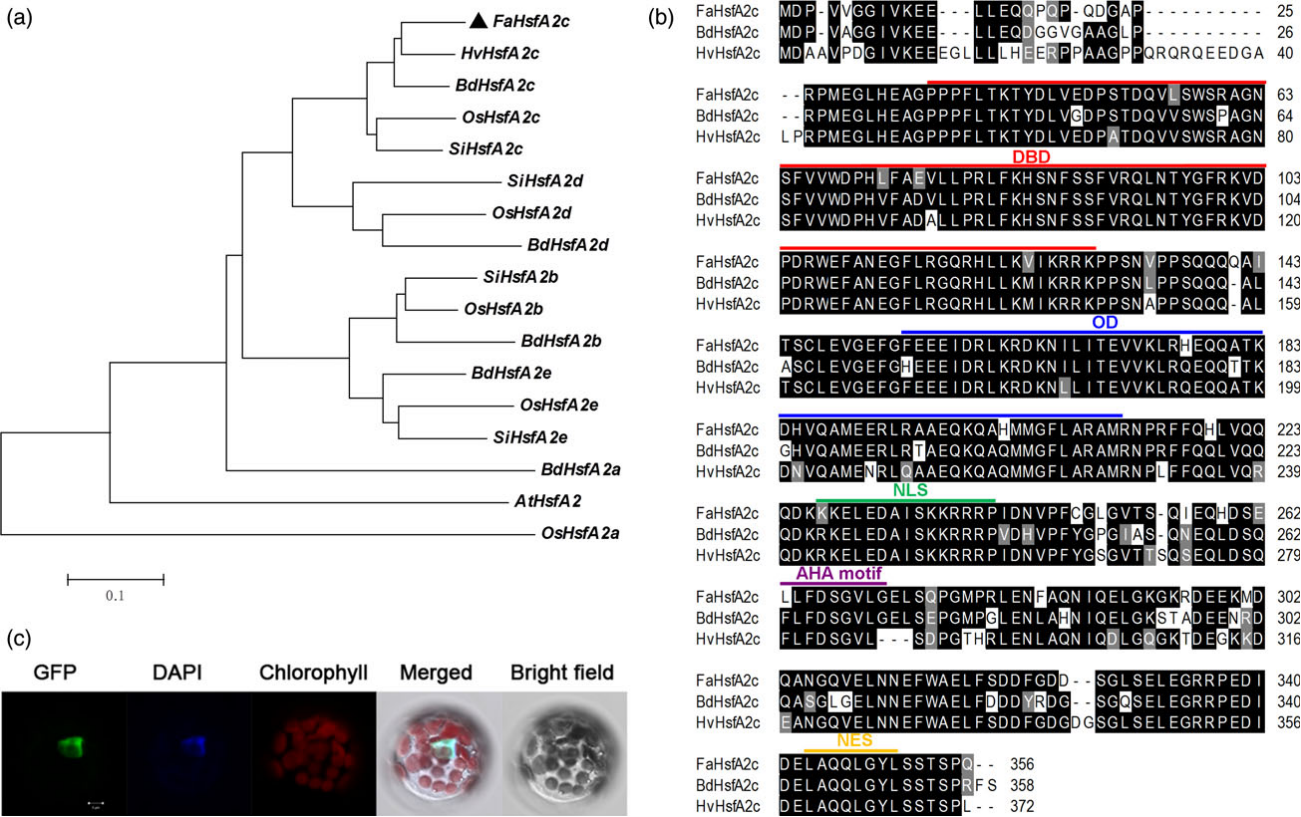
Identification of FaHsfA2c. (a) Phylogenetic relationships between FaHsfA2c and 16 additional orthologs. The phylogenetic tree was constructed in MEGA 4 (Tamura *et al*., 2007) using the neighbour‐joining method. (b) Amino acids sequence comparison and conserved domains of FaHsfA2c and its closest orthologs *Brachypodium distachyon* HsfA2c and *Hordeum vulgare* HsfA2c. Sequence alignment was performed with ClustalW Multiple alignment in BioEdit. Black regions indicate identical amino acids among the three sequences; grey regions indicate similar amino acids. DBD, DNA‐binding domain; OD, oligomerization domain; NLS, nuclear localization signal; AHA motif, activator motif; NES, nuclear export signal. (c) Subcellular localization of FaHsfA2c. FaHsfA2c‐GFP fusion protein was localized in nucleus of *Arabidopsis* mesophyll protoplast. Bar = 5 μm.

According to the assembled sequence of *HsfA2* in tall fescue EST database (Saha *et al*., 2004), one 1102‐bp homologous sequence was isolated from cDNA library of ‘Barlexas’. This sequence was clustered into the A2c category of *HsfA2* group and showed the highest homology to *Hordeum vulgare HsfA2c* according to the phylogenetic analysis (Figure 1a), which was designated as *Festuca arundinacea HsfA2c* (*FaHsfA2c*). *FaHsfA2c* includes a 1068‐bp open reading frame predicted to encode 356 amino acids that showed high sequence identity with *HvHsfA2c* and *Brachypodium distachyon HsfA2c* (*BdHsfA2c*; Figure 1b). Additionally, analysis of *FaHsfA2c* polypeptide structure revealed the presence of a DNA‐binding domain (DBD) located close to the N‐terminus of FaHsfA2c, an OD or HR‐A/B region adjacent to the DBD, an activator motif (AHA motif) and a nuclear export signal (NES) domain located close to the C‐terminus.

The predicted nuclear localization signal (NLS) domain between OD and AHA motif represents a putative nuclear targeting of FaHsfA2c. To verify its subcellular localization, a *GFP*‐fused *FaHsfA2c* was constructed and transiently transformed into *Arabidopsis* protoplasts. The aligned fluorescence of GFP and DAPI, a dye specially stains nuclei, indicated the nuclear localization of FaHsfA2c (Figure 1c).

### Expression patterns of tall fescue A2‐type *HSFs* in HSR

**Figure 2:**
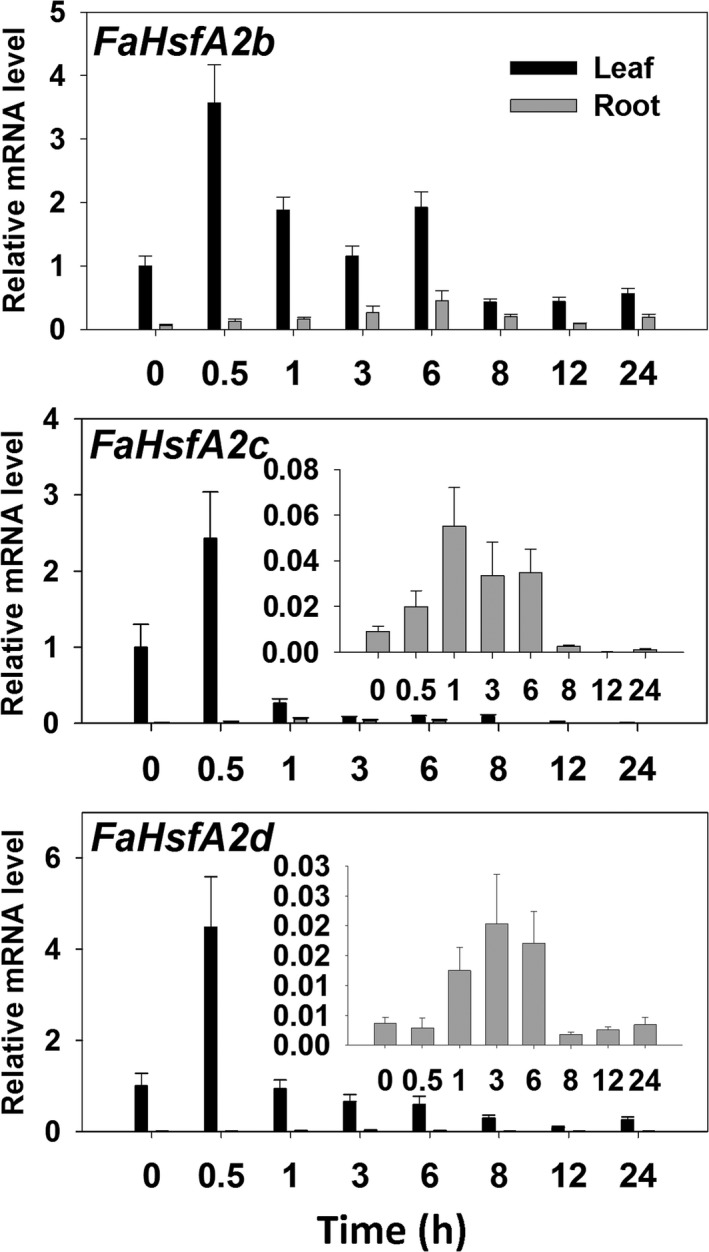
Expression patterns of *FaHsfA2b*,* c* and *d* in response to heat. Tall fescue was treated at 37 °C for 6 h followed by recovery at 25 °C. qRT‐PCR data are expressed as the mean values ± standard deviation (SD) of three biological replicates.

To identify the expression pattern of different A2‐type members of *HSFs* in tall fescue, three A2‐type genes (*FaHsfA2b*,* c* and *d*) obtained from the tall fescue EST database were analysed using qRT‐PCR. The transcription levels of all the three genes increased and reached to the peak by 2.5‐ to 5‐fold within 30 min and then decreased rapidly in leaves under 37 °C HS (Figure 2). However, in roots, the expression of *FaHsfA2b* exhibited a gradual increase within 6 h of heat treatment while *FaHsfA2c* and *FaHsfA2d* reached the highest level within 1 and 3 h of HS, respectively. Overall, the transcripts of the three genes in leaves were higher than in roots regardless of temperature treatments. After the transient increase, the transcription levels of all three genes attenuated with extended heat stress duration. In the recovery stage following 6 h of HS, transcripts of all three genes decreased to the initial levels in both leaves and roots.

### FaHsfA2c conferring heat tolerance in *Arabidopsis*

**Figure 3:**
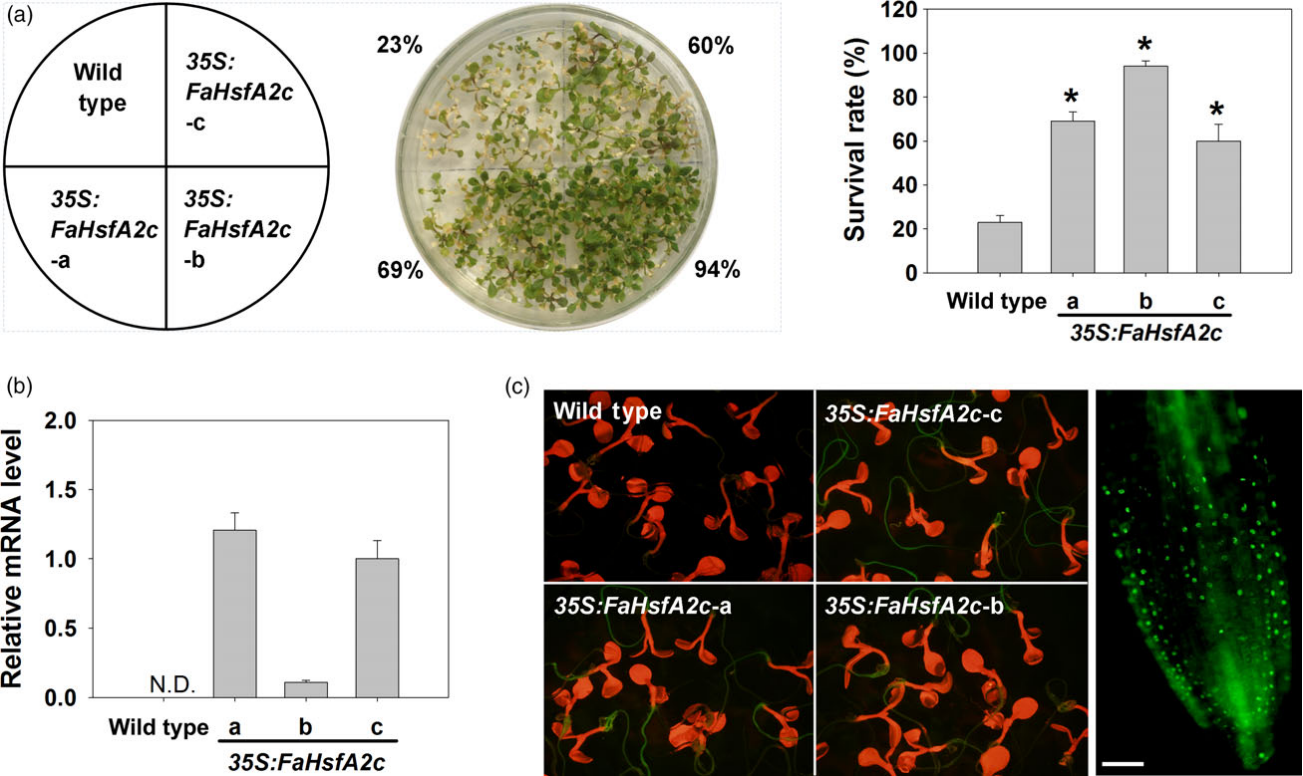
Ectopic expression of *FaHsfA2c* in wild‐type *Arabidopsis*. (a) Selection of heat‐tolerant transgenic lines. Thirty seeds of each selection were grown on agar medium at 25 °C for 7 day and then placed into growth chamber maintained at 45 °C for 4 h. The photograph was taken 7 day after heat treatment. Values and the column chart indicate the total survival rate of plants in three petri dishes for each line. Bars represent ± SD. Asterisk (*) indicates significant difference between each transgenic line and wild type according to Fisher's protected LSD test at a significance level of 0.05. (b) *FaHsfA2c* expression levels in wild‐type *Arabidopsis* and transgenic lines (*35S:FaHsfA2c‐*a, b and c). N.D., not detected. (c) Expression of FaHsfA2c‐GFP fusion protein in *35S:FaHsfA2c* transgenic lines. Five‐day‐old seedlings on agar plate were observed under stereo fluorescence microscope (left panel). Root tips of transgenic lines were scanned with higher magnification of microscopy (right panel). Bar = 50 μm.

*FaHsfA2c* was further analysed for the examination of its functions in regulating heat tolerance, as the physiological functions of HsfA2c related to heat tolerance has not been investigated even though it has been found in rice and barley. *FaHsfA2c* with GFP fusion was constitutively expressed in *Arabidopsis* under the control of *CaMV35S* promoter. Three transgenic lines (*35S:FaHsfA2c‐a*, ‐*b* and ‐*c*) that exhibited significantly higher survival rates (69%, 94% and 60%) than the wild type (23% survival rate) under 45 °C heat stress were chosen for further physiological analysis (Figure 3a). Ectopic expression of FaHsfA2c in *Arabidopsis* did not lead to any phenotypic variation compared to the wild type. The transcripts of *FaHsfA2c* were detected in all three *35S:FaHsfA2c* transgenic lines but not in the wild type, indicating successful transformation (Figure 3b). GFP‐fused FaHsfA2c revealed bright green fluorescence in roots of three transgenic lines although it was not obvious in leaves due to the interference by chlorophyll fluorescence (Figure 3c, left panel), and the enlarged view of the root tips of transgenic plants was displayed (Figure 3c, right panel).

**Figure 4:**
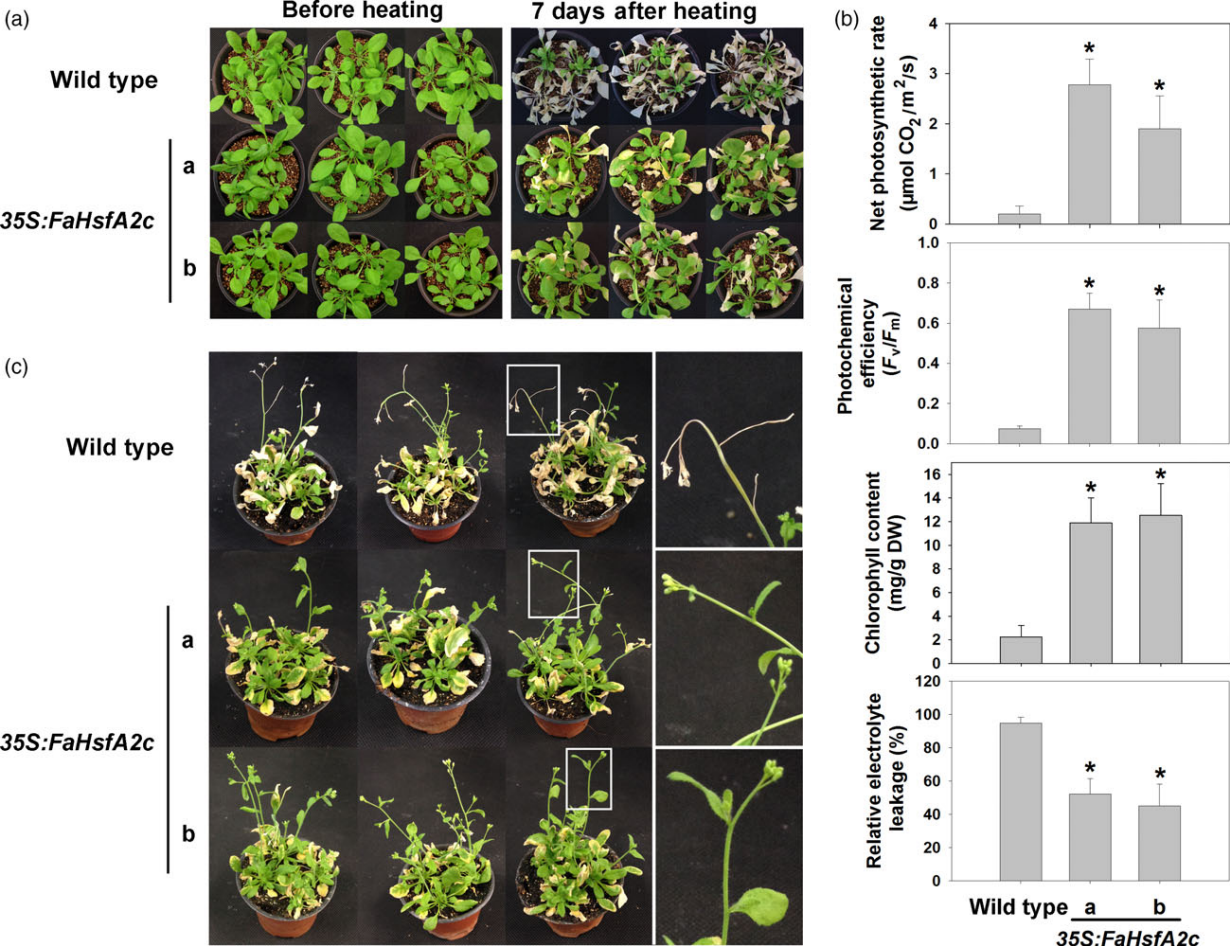
Basal and acquired thermotolerance of *35S:FaHsfA2c* transgenic lines. (a) Phenotype of 30‐day‐old wild type and *35S:FaHsfA2c* transgenic plants before heat stress of 45/40 °C (day/night) for 72 h and after 7 day recovery at 25 °C. (b) Net photosynthetic rate, photochemical efficiency, chlorophyll content and relative electrolytic leakage of plants in (a) were measured after recovery. Data are expressed as the mean values ± SD of three biological replicates. Asterisk (*) indicates significant difference between each transgenic line and wild‐type plants according to Fisher's protected LSD test at a significance level of 0.05. (c) Phenotype of 30‐day‐old wild‐type and *35S:FaHsfA2c* transgenic plants preheated at 37 °C for 2 h followed by 25 °C for 2 day, then put into 45/40 °C (day/night) conditions for 3.5 day. Photographs were took after 7 day recovery at 25 °C. Details of inflorescence marked in the white boxes were amplified on the right side.

**Figure 5:**
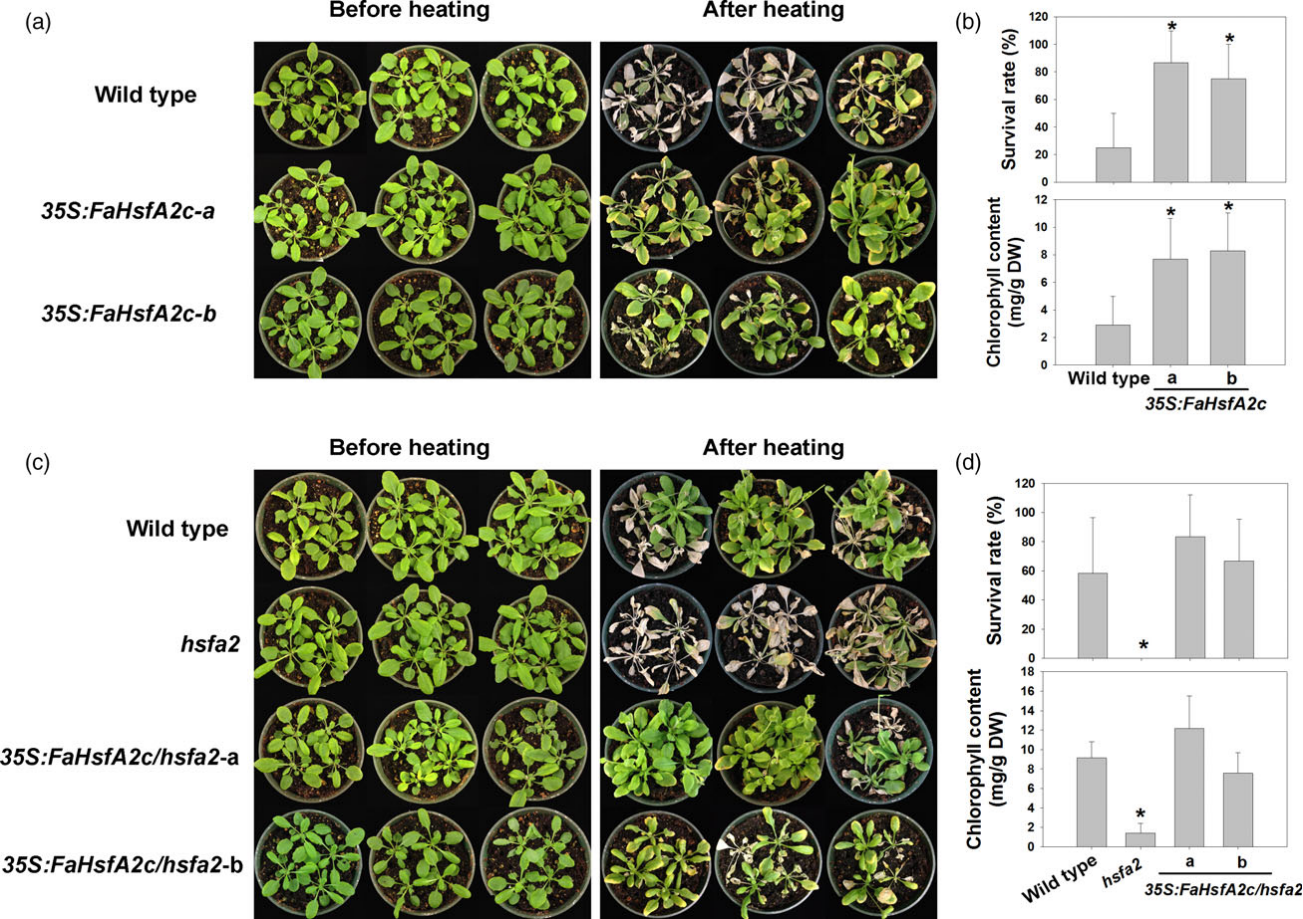
Characteristic of transgenic *Arabidopsis* under heat stress at 37/32 °C (day/night). (a) Phenotype and (b) survival rate and chlorophyll content of wild‐type *Arabidopsis* and *35S:FaHsfA2c* transgenic lines after 12 day of heat treatment. Asterisk (*) indicates significant difference between each transgenic line and wild type according to Fisher's protected LSD test at a significance level of 0.05. (c) Phenotype and (d) survival rate and chlorophyll content of wild‐type *Arabidopsis, hsfa2* mutant and *35S:FaHsfA2c/hsfa2* transgenic lines after 9 day of heat treatment. Asterisk (*) indicates significant difference between *hsfa2* and others according to Fisher's protected LSD test at a significance level of 0.05.

Physiological responses of plants from heat stress were compared between the wild‐type and two transgenic *Arabidopsis* lines (*35S:FaHsfA2c*‐a and *35S:FaHsfA2c*‐b), which showed higher thermotolerance in Figure 3a. After 7 day of exposure to the ambient temperature following 3 day of severe heat stress at 45/40 °C (day/night), leaves of the wild type remained wilted and did not show any sign of recovery, while both transgenic lines exhibited significant recovery with only a few yellow leaves remaining (Figure 4a). Leaf net photosynthetic rate, photochemical efficiency and chlorophyll content of both transgenic lines were significantly higher than those of the wild type (Figure 4b). And the relative electrolyte leakage, as an indicator of membrane stability, was about 50% lower in the two transgenic lines than that of the wild type. In the acquired thermotolerance assay, transgenic plants also recovered better after heat stress and had fewer yellow leaves than the wild type (Figure 4c). The top of the inflorescence stem of the wild type wilted while that of the transgenic plants had no sign of heat damages. Similarly, after 12 day of moderate heat stress at 37/32 °C (day/night), transgenic plants showed higher survival rates and chlorophyll contents than the wild type (Figure 5a and b).

**Figure 6:**
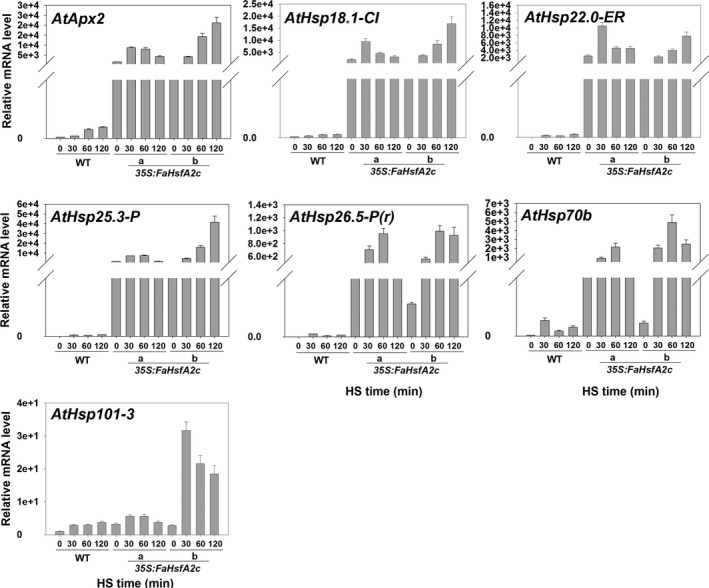
Expression patterns of potential target genes of FaHsfA2c. Leaves of wild‐type and two transgenic lines were sampled at 0, 30, 60 and 120 min during heat treatment at 37 °C for qRT‐PCR. HS, heat stress. Data are expressed as the relative mean values ± SD of three biological replicates.

Expression patterns of downstream genes of *FaHsfA2c* in response to heat stress were examined and compared between the wild‐type and two transgenic lines (*35S:FaHsfA2c*‐a and *35S:FaHsfA2c*‐b) using qRT‐PCR. Before heat treatment, the transcripts of *AtApx2*,* AtHsp18.1‐CI*,* AtHsp22.0‐ER*,* AtHsp25.3‐P*,* AtHsp26.5‐P(r)*,* AtHsp70b* and *AtHsp101‐3* in *35S:FaHsfA2c*‐a and *35S:FaHsfA2c*‐b were all higher than those in the wild type due to constitutively expressed *FaHsfA2c* (Figure 6). The expression levels of all those genes were up‐regulated in response to the heat treatment in the wild‐type and transgenic plants. The expression patterns of the downstream genes in HSR were similar to heat‐response patterns of *FaHsfA2c*, as described above. However, the transcription levels of all genes were significantly higher in transgenic lines than that in the wild type.

### FaHsfA2c rescue of heat‐sensitive defects of *Arabidopsis hsfa2* mutant

**Figure 7:**
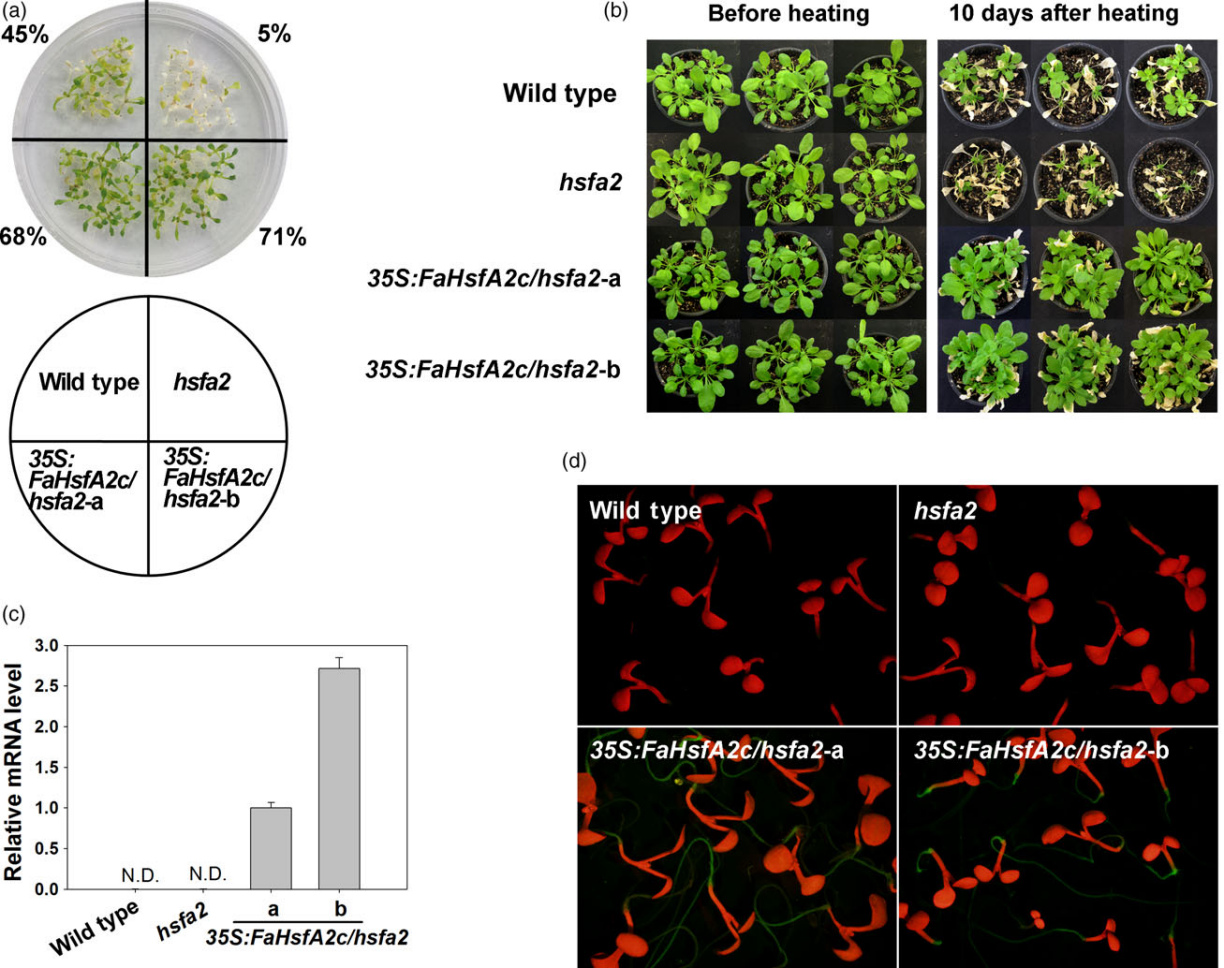
Complementary assay of *Arabidopsis hsfa2* mutant with transformed *FaHsfA2c*. (a) Selection of heat‐tolerant transgenic lines. The experiments were performed as described in Figure 3a, except that 16 seeds for each line were grown on one petri dish and four petri dishes as replicates were treated at 45 °C for 3 h. Values indicate the total survival rate of plants in four petri dishes for each line. (b) Heat tolerance of wild‐type *Arabidopsis, hsfa2* mutant and transgenic lines (*35S:FaHsfA2c/hsfa2*‐a and ‐b). The 30‐day‐old plants were treated at 45/40 °C (day/night) for 60 h and then recovered at 25 °C for 10 day. (c) FaHsfA2c expression levels in wild‐type *Arabidopsis*,* hsfa2* mutant and transgenic lines. N.D., not detected. (d) Expression of FaHsfA2c‐GFP fusion protein in *35S:FaHsfA2c/hsfa2* transgenic lines. Five‐day‐old seedlings on agar plate were observed under stereo fluorescence microscope.

To further address FaHsfA2c functions in regulating heat tolerance, *GFP*‐fused *FaHsfA2c* with *CaMV35S* was also introduced into *Arabidopsis hsfa2* mutant, which is sensitive to heat stress. Two transgenic lines (*35S:FaHsfA2c/hsfa2*‐a and *35S:FaHsfA2c/hsfa2*‐b) with significantly higher survival rate (68% and 71%) than wild type (45%) and *hsfa2* mutant (5%) under heat stress were selected for further analysis (Figures 7a and S1a). *Arabidopsis hsfa2* mutant plants became desiccated and died after severe heat stress of 45/40 °C (day/night) for 60 h (Figure 7b) or moderate heat stress of 37/32 °C (day/night) for 9 day (Figure 5c). However, overexpression of *FaHsfA2c* restored the heat‐sensitive defects of *hsfa2* mutant with higher survival rates or chlorophyll contents of *35S:FaHsfA2c/hsfa2*‐a and *35S:FaHsfA2c/hsfa2*‐b than the *hsfa2* and the wild type after heat stress (Figures 5d and S1b). The transcripts (Figure 7c) and GFP‐fused protein (Figure 7d) of FaHsfA2c were detected in transgenic lines, but not in the wild type and *hsfa2* mutant.

### Overexpression of *FaHsfA2c* conferring heat tolerance in tall fescue

**Figure 8:**
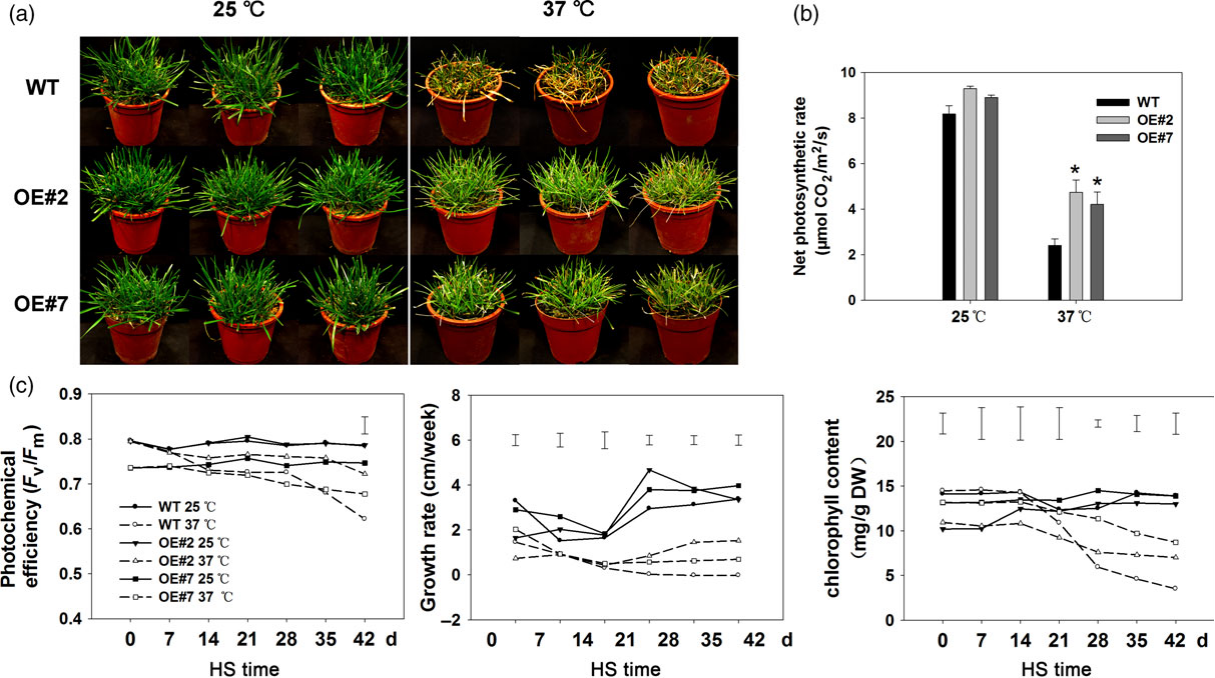
Physiological analyses of tall fescue plants overexpressing *FaHsfA2c* after heat stress. (a) Effects of heat treatment (37/32 °C) on wild‐type tall fescue and transgenic lines (OE#2 and OE#7) for 42 day. Plants at 25/20 °C as the parallel control. Three pots represent three biological replicates. (b) Net photosynthesis rates of wild‐type and transgenic tall fescue under 25 °C and 37 °C for the 42 day. Bars represent ± SD. Asterisk (*) indicates significant difference between each transgenic line and wild type under heat stress according to Fisher's protected LSD test at a significance level of 0.05. (c) Characterization of photochemical efficiency, growth rate and chlorophyll content of wild‐type and *FaHsfA2c* transgenic tall fescue during 42 day of heat stress and control conditions. HS, heat stress. Vertical bars represent LSD values among the data below at *P *=* *0.05.

After 42 day of heat treatment at 37/32 °C (day/night), most of the wild‐type tall fescue died but some transgenic lines overexpressing *FaHsfA2c* remained alive, from which two transgenic lines (OE#2 and OE#7) with higher thermotolerance were selected for further analysis (Figure 8a). Overexpression of *FaHsfA2c* in tall fescue did not lead to any remarkable changes in plant morphology. The transcripts of *FaHsfA2c* in OE#2 and OE#7 were enhanced under the control of *CaMV35S* promoter (Figure S2). Net photosynthetic rate in all the plants declined after heat stress, to a greater degree in the wild type (Figure 8b). Photochemical efficiency in all heat‐treated plants declined throughout the 42 day of heat stress, and by 28 day, photochemical efficiency decreased by 4.2% and 4.9% in OE#2 and OE#7, respectively, while it decreased by 8.9% in wild‐type plants (Figure 8c). After 42 day of heat treatment, transgenic lines had significantly higher photochemical efficiency compared to the wild type. Chlorophyll content also declined in all heat‐treated plants during heat stress, to a greater degree in the wild type. Transgenic plants maintained significant higher chlorophyll content compared to the wild‐type plants at 28, 35 and 42 day of heat stress. The growth rate of wild type was also obviously lower than transgenic plants after 28 day of heat treatment.

**Figure 9:**
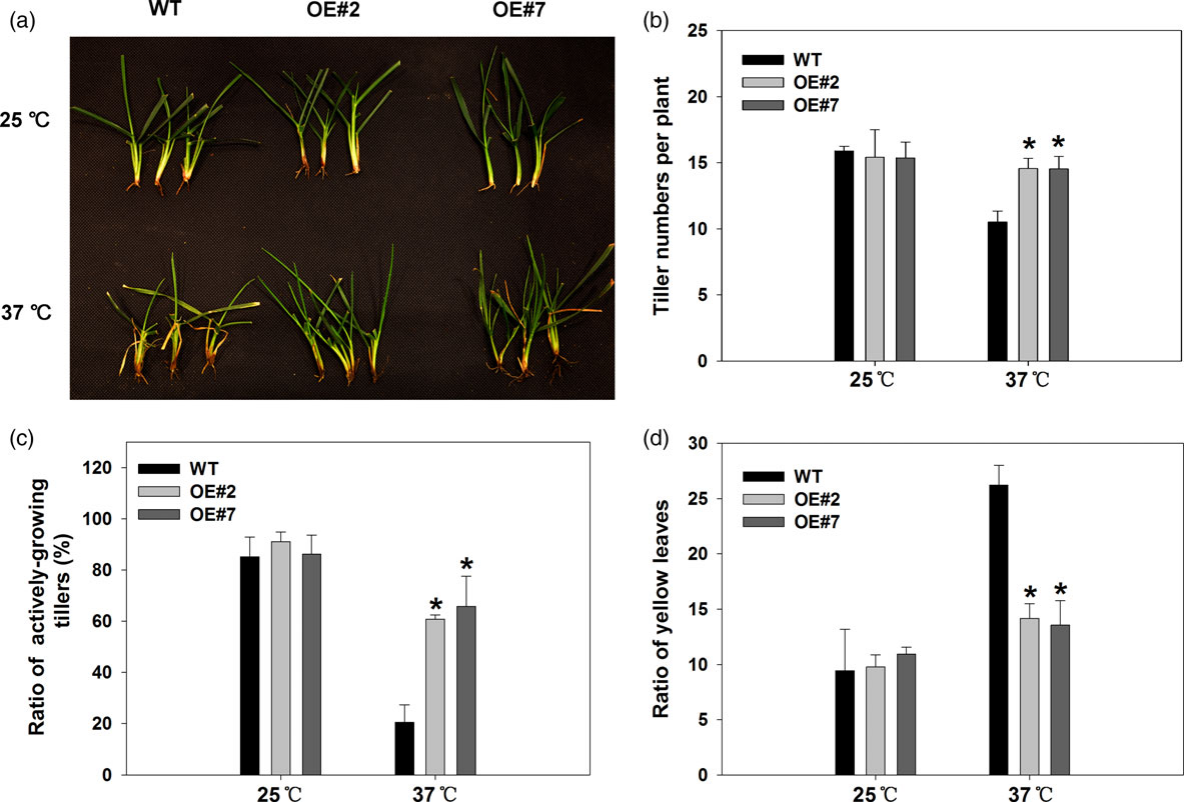
Effects of *FaHsfA2c* overexpression on tall fescue growth or phonotypic traits at 42 day of heat stress and control conditions: (a) Phenotype of detailed tillers; (b) tiller numbers; (c) ratio of actively growing tillers and (d) ratio of yellow leaves. These parameters were based on all the tillers or leaves of eight plants in one pot, and three pots for each line were calculated. Bars represent ± SD. Asterisk (*) indicates significant difference between each transgenic line and wild type under heat stress according to Fisher's protected LSD test at a significance level of 0.05.

The photograph of tillers of the wild‐type and transgenic plants overexpressing *FaHsfA2c* depicted that the wild type showed greater degree of injury compared to OE#2 and OE#7 under heat stress (Figure 9a). The less degree of heat damages in the transgenic tall fescue than the wild type were also manifested by greater tiller numbers (Figure 9b), ratio of actively growing tillers (Figure 9c) and lower ratio of yellow leaves in the transgenic lines (Figure 9d). Tiller growth of the wild type was significantly inhibited under heat stress, while that of transgenic plants was almost not affected. Long‐term high temperature also suppressed tiller vitality and led to more yellow leaves in all the heat‐treated plants, to a greater degree in the wild type.

### Regulatory roles of FaHsfA2c mediating multiple HSP response in tall fescue

**Figure 10:**
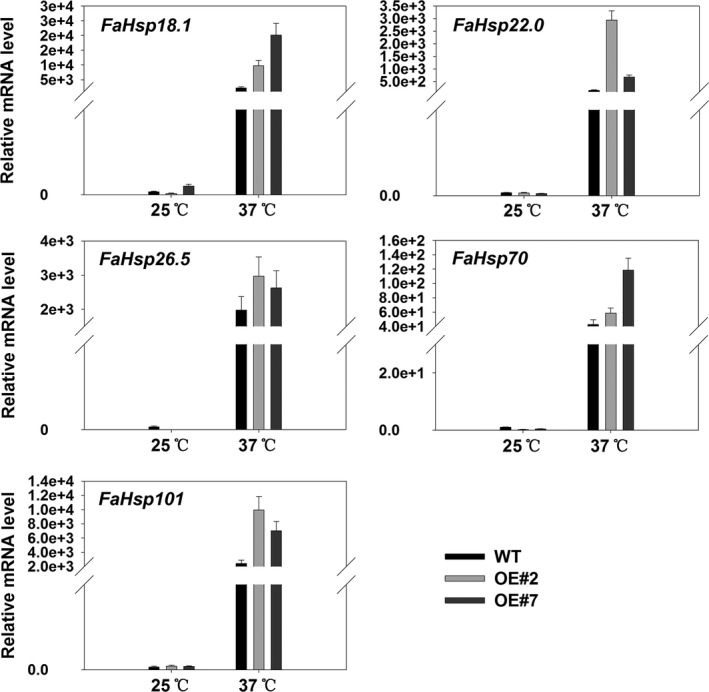
qRT‐PCR analysis of candidate genes differentially expressed between wild‐type and transgenic tall fescue overexpressing *FaHsfA2c* at 25 °C or 37 °C for 1 h. Data are expressed as the mean values ± SD of three biological replicates.

In consideration of the regulatory roles of FaHsfA2c for *AtApx2*,* AtHsp18.1‐CI*,* AtHsp22.0‐ER*,* AtHsp25.3‐P*,* AtHsp26.5‐P(r)*,* AtHsp70b* and *AtHsp101‐3* in *Arabidopsis*, their homologous genes in tall fescue were analysed, except *Hsp25.3,* for which the sequence is not available in tall fescue database*. FaHsp18.1*,* FaHsp22.0*,* FaHsp26.5*,* FaHsp70* and *FaHsp101* were up‐regulated by 1 h of 37 °C heat stress relative to that of 25 °C (Figure 10). Transcription levels of these genes in OE#2 and OE#7 were significantly higher than that in the wild type under heat stress. However, *FaApx2* was not induced by 1 h or even 3 h of heat stress in tall fescue (data not shown), which exhibited different expression patterns from that in *Arabidopsis*.

## Discussion

In this study, three A2‐type *HSFs* were first identified in a cool‐season perennial grass species. Sequence analysis and subcellular localization of *FaHsfA2c* confirmed it is a member of HSF located in nucleus. *HsfA2c* has also been annotated in barley (*HvHsfA2c*) and *Brachypodium* (*BdHsfA2c*) with genome sequence released in NCBI, which show high similarity to *FaHsfA2c* as found in this study. However, the physiological functions of BdHsfA2c have not been previously reported, and HvHsfA2c was mentioned to be responsive to heat in barley caryopses, but its functions in regulating heat tolerance has yet to be determined (Mangelsen, 2010). In this study, it was found that FaHsfA2c possesses highly structured DBD, which contains a hydrophobic core that ensures the precise positioning and selective interaction with heat‐shock elements (HSEs) of targeted genes, the AHA motif that only exists in class A HSFs responsible for transcriptional activation on the targets, and the NLS and NES domains make sure the subcellular distribution of FaHsfA2c between nucleus and cytoplasm (Lin *et al*., 2011). The OD in FaHsfA2c is crucial for oligomerization, an activated state that is conducive to form super‐activated heterooligomers with HsfA1 (Scharf *et al*., 2012). These key domains and characteristics of the gene structure ensure for maintaining the effective function of FaHsfA2c in mediating HSR.

Plant adaptation to high‐temperature environments requires transcriptional reprogramming of a series of genes involved in heat stress protection (Xue *et al*., 2015). *HSPs* are a family of target genes of HSF, some of which are known to act as chaperones preventing misfolding and aggregation of other proteins during heat stress (von Koskull‐Doering *et al*., 2007). In our study, multiple *HSP* genes, including *sHSPs*,* Hsp70* and *Hsp101*, were up‐regulated to a greater degree by overexpression of *FaHsfA2c* both in *Arabidopsis* and tall fescue compared to the respective wild type, indicating that FaHsfA2c could regulate those *HSPs* in transgenic plants. Previous studies in *Arabidopsis* reported that HsfA2 may also regulate the expression of *Apx2* related to oxidative stress (Schramm *et al*., 2006). In this study, *Apx2* expression was also up‐regulated by ectopic‐expressed FaHsfA2c in *Arabidopsis* but not affected in tall fescue overexpressing *FaHsfA2c* under heat stress. These results suggested that the regulation of HSR by FaHsfA2c in tall fescue may not involve *Apx2* unlike that in *Arabidopsis* and also implied that FaHsfA2c mediated different molecular components in the cool‐season grass species from *Arabidopsis*. Other members of FaHsfA2s rather than FaHsfA2c in tall fescue perhaps regulate *Apx2* because that the specific HsfA2s with functions of regulating HSR may vary with plant species that exhibit differential HSR.

Physiological analysis of transgenic plants overexpressing or ectopically expressing *FaHsfA2c* revealed that it positively regulated heat tolerance in both tall fescue and *Arabidopsis* in this study. Photosynthetic activity is among the most heat‐sensitive physiological processes in plant cells (Wahid *et al*., 2007). Net photosynthetic rate declined with heat stress, but maintained at significantly higher level in plants overexpressing *FaHsfA2c*, suggesting that *FaHsfA2c* plays positive roles in regulating photosynthesis responses to heat stress. The chloroplast small heat‐shock proteins (Cp‐sHSP) including many members ranging from 21 to 26 kDa are transported from the cytoplasm to the chloroplast in high‐temperature conditions to protect chloroplast stroma from oxidative stress and facilitate the components refolding of photosystem II (PSII) during heat stress (Chauhan *et al*., 2012; Ul Haq *et al*., 2013). PSII has been considered as the primary site of heat impairments of photosynthesis, and the most efficient tools for monitoring heat effects on PSII are based on the photochemical efficiency recording (Brestic and Zivcak, 2013). The transgenic lines overexpressing *FaHsfA2c* exhibited higher photochemical efficiency than the wild type for both *Arabidopsis* and tall fescue, indicating FaHsfA2c could have protective roles for PSII. Damage of PSII will affect photosynthetic electron transfer to PSI and then lead to decrease of NADPH supply for CO_2_ fixation (Brestic *et al*., 2014), subsequently influence carbohydrate synthesis and finally affect plant growth and development. Chlorophyll degradation and loss of membrane stability are also typical heat stress damages of grass species (Li *et al*., 2013). In this study, both chlorophyll and membrane stability decreased during heat stress, but transgenic plants overexpressing *FaHsfA2c* exhibited higher levels of both parameters, indicating that FaHsfA2c could affect chloroplast or thylakoid membrane integrity with facilitating chlorophyll retention and membrane stability under heat stress.

In summary, *FaHsfA2c* was first cloned, characterized and functionally analysed in perennial grass species associated with heat tolerance. Structure analysis demonstrated that FaHsfA2c possesses all key domains and characteristics of HSF for proper regulatory function for HSR. The positive effects of FaHsfA2c in regulating heat tolerance were confirmed through growth and physiological analysis of plants overexpressing the gene in tall fescue and wild‐type *Arabidopsis* and restoring the heat‐sensitive deficiency of *hsfa2* mutant. The analysis of downstream genes regulated by FaHsfA2c demonstrated that it could positively regulate heat tolerance due to the high‐efficient transcriptional regulation of downstream genes involved in heat response and protein protection. Further studies will characterize the promoter of *FaHsfA2c* and find the potential signal factors for activating FaHsfA2c‐mediated heat tolerance in tall fescue. *FaHsfA2c* could be used as a candidate gene to improve heat tolerance of cool‐season grass species through genetic engineering and developing molecular markers assisting breeding selection of heat‐tolerant grass germplasm.

## Experimental procedures

### Plants growth conditions and heat treatment

Tall fescue (cv. Barlexas) seeds were geminated on filter paper soaked in sterile water in a petri dish, which were transferred to pots filled with peat–vermiculite mix (3 : 1 v/v) in a growth chamber at 25/20 °C (day/night) with a 12‐h photoperiod of photosynthetically active radiation of 650 μmol photons/m^2^/s. For gene isolation and expression, 1‐month‐old plants were transferred to a hydroponic system with half‐strength Hoagland's nutrient solution (Hoagland and Arnon, 1950) and grown for additional 3 weeks before heat treatment. Plants were initially exposed to 37 °C for 6 h of heat stress treatment and then treated at 25 °C to examine recovery. Leaves and roots were sampled at the time points of 0, 0.5, 1, 3, 6, 8, 12 and 24 h.

For thermotolerance selection of transgenic tall fescue, 1‐month‐old plants regenerated from tissue culture were transferred to pots filled with peat–vermiculite mix (3 : 1 v/v) to allow plant establishment for additional 2 months. Then, plants were subjected to control temperature at 25/20 °C (day/night) or heat stress at 37/32 °C with a 14‐h photoperiod for 42 days, and leaves were collected every week for analysis.

*Arabidopsis* ecotype Colombia was used as the wild type, and the *HsfA2* (At2 g26150) T‐DNA insertion mutant SALK_008978 (*hsfa2*) (Alonso, 2003) was obtained from the *Arabidopsis* Biological Resource Center (ABRC; Ohio State University), with homozygous mutants confirmed by PCR. Sterilized seeds of T1 and T2 heterogeneous transgenic *Arabidopsis* were geminated in Murashige and Skoog (MS) medium with glufosinate ammonium (20 mg/L) at 25/20 °C (day/night) with a 12‐h photoperiod while seeds of T3 homozygous transgenic plants, wild type and *hsfa2* mutant were germinated in Murashige and Skoog (MS) medium without glufosinate ammonium. T3 seedlings on plates were used for selection of thermotolerant lines and protein localization by green fluorescent protein (GFP) observation. Another set of seedlings on plates was transferred to plastic pots filled with peat–vermiculite mix (3 : 1 v/v) to propagate plants for further examination of heat responses of mature plants and for gene expression analysis. To evaluate physiological responses to heat stress, wild type, *hsfa2* mutant and *FaHsfA2c* transgenic plants were exposed to heat stress at 45/40 °C (day/night) or 37/32 °C (day/night).

### Gene isolation of *FaHsfA2c* and qRT‐PCR analysis

Total RNA was extracted from heat‐treated (37 °C for 1 h) tall fescue leaves, and cDNA was synthesized as template for *FaHsfA2c* isolation, with primers FaHsfA2c‐F and FaHsfA2c‐R designed according to assembled EST sequence of tall fescue (Saha *et al*., 2004). *FaHsfA2c* was confirmed by sequencing and blast on NCBI (National Center for Biotechnology Information, http://www.ncbi.nlm.nih.gov/).

For qRT‐PCR analysis, total RNA of tall fescue or *Arabidopsis* was extracted using RNApure fast isolation Kit (YuanPingHao, Tianjin, China) and first‐strand cDNA was synthesized using the PrimeScript RT reagent Kit with gDNA Eraser (Perfect Real Time) (Takara, Otsu, Japan). PCRs were performed in triplicate with SYBR Green I Master reaction system (Roche Diagnostic, Rotkreuz, Switzerland) on Roche LightCycler480 II (Roche Diagnostic, Rotkreuz, Switzerland). Data were normalized according to the *AtActin2* (for *Arabidopsis*) or *FaEF1*α (for tall fescue) gene expression levels and determined by 2^−ΔΔCT^ calculation methods. Primers for qRT‐PCR are listed in Table S1.

### Plasmid construction and plant transformation

The ORF of *FaHsfA2c* with *Sal* I and *Not* I sites was first introduced into a pENTR1A Dual Selection Vector and then transformed into a pEarleyGate 103 (Earley *et al*., 2006) destination plasmid. The destination plasmid in *Agrobacterium tumefaciens* strain EHA105 was transformed into *Arabidopsis* using the floral dip method (Clough and Bent, 1998) and into tall fescue through calli infection (Wang and Ge, 2005). The positive transgenic lines of *Arabidopsis* and tall fescue were selected through glufosinate ammonium resistance and PCR confirmation.

For subcellular localization, the pENTR1A Vector containing *FaHsfA2c* was LR‐recombined with a small binary vector p2GWF7.0, which contains an enhanced green fluorescent protein (eGFP). Then, the construct was transformed into *Arabidopsis* protoplasts for observation of eGFP‐fused FaHsfA2c. Isolation, purification and PEG‐mediated transformation of *Arabidopsis* protoplasts were performed as previously described (Wu *et al*., 2009). After incubation for 16 h, the transfected protoplasts were mixed with DAPI solution to a final concentration of 10 μg/mL for a 5‐min incubation at room temperature and then observed under a confocal laser scanning microscope (Carl Zeiss, Jena, Germany). For the transgenic *Arabidopsis*, root tips of 3‐day‐old seedlings were cut and placed on slides for GFP observation using a fluorescence microscope BX53 (Olympus, Tokyo, Japan).

### Physiological measurements of transgenic *Arabidopsis* and tall fescue

Leaf net photosynthetic rate (Pn) was determined using a LI‐6400 portable photosynthesis system (LI‐COR, Lincoln, NE, USA) and leaf photochemical efficiency was conducted with a fluorescence induction monitor (OPTI‐Sciences, Hudson, USA). Chlorophyll content of leaves was measured using a dimethyl sulfoxide extraction method, and membrane stability of leaves was estimated by measuring electrolyte leakage (Jespersen and Huang, 2015). Growth rate was presented as canopy height every week. Tiller numbers per plant was presented as means of 10 plants for each replicate. Ratio of actively growing tillers was calculated as the ratio of the number of tillers that actively generated new leaves to the total number of tillers per plant. Ratio of yellow leaves was calculated as the ratio of the number of leaves with at least half of the leaf length exhibiting chlorosis to the number of total leaves per plants.

### Statistical analyses

Heat stress effects and variations among wild type, mutants and transgenic plants for physiological parameters and gene expression levels were analysed using the ANOVA model with PASW statistics 18 (SPSS Inc, Chicago, IL, USA). Mean data were separated using Fisher's protected least significance difference (LSD) at a significance level of 0.05.

## Conflict of interest

There is no conflict of interest to declare.

## Supporting information


**Figure S1** Survival rates of wild‐type *Arabidopsis, hsfa2* mutant and *35S:FaHsfA2c/hsfa2* transgenic lines in (a) Figure 7a and (b) Figure 7b. Bars represent ± SD. Asterisk (*) indicates significant difference between *hsfa2* and others according to Fisher's protected LSD test at a significance level of 0.05.

**Figure S2** Relative gene expression level of *FaHsfA2c* in wild‐type tall fescue and transgenic lines. Bars represent ± SD. Asterisk (*) indicates significant difference between each transgenic line and wild type according to Fisher's protected LSD test at a significance level of 0.05.

**Table S1** Primers used in the study.
