# Supplementary figures and images for: Molecular regulation and physiological functions of a novel FaHsfA2c cloned from tall fescue conferring plant tolerance to heat stress
Source: Plant Biotechnol J. 2016 Sep 23;15(2):237–48. doi: 10.1111/pbi.12609 (PMC5258862; doi:10.1111/pbi.12609)

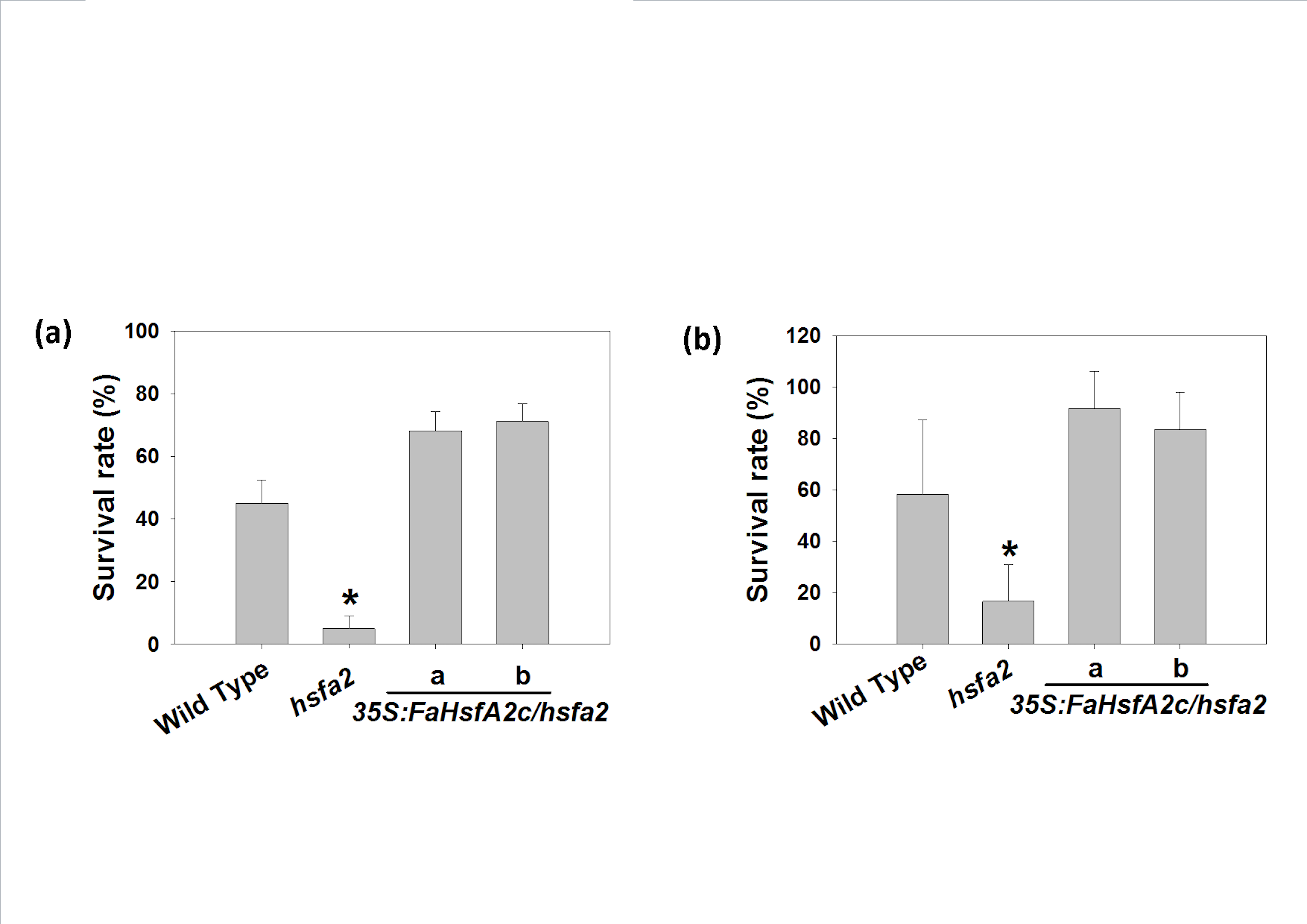

Supplement: Supplementary file 1 — Figure S1 Survival rates of wild‐type Arabidopsis, hsfa2 mutant and 35S:FaHsfA2c/hsfa2 transgenic lines in (a) Figure 7a and (b) Figure 7b. Bars represent ± SD. Asterisk (*) indicates significant difference between hsfa2 and others according to Fisher's protected LSD test at a significance level of 0.05. [file PBI-15-237-s003.tif]

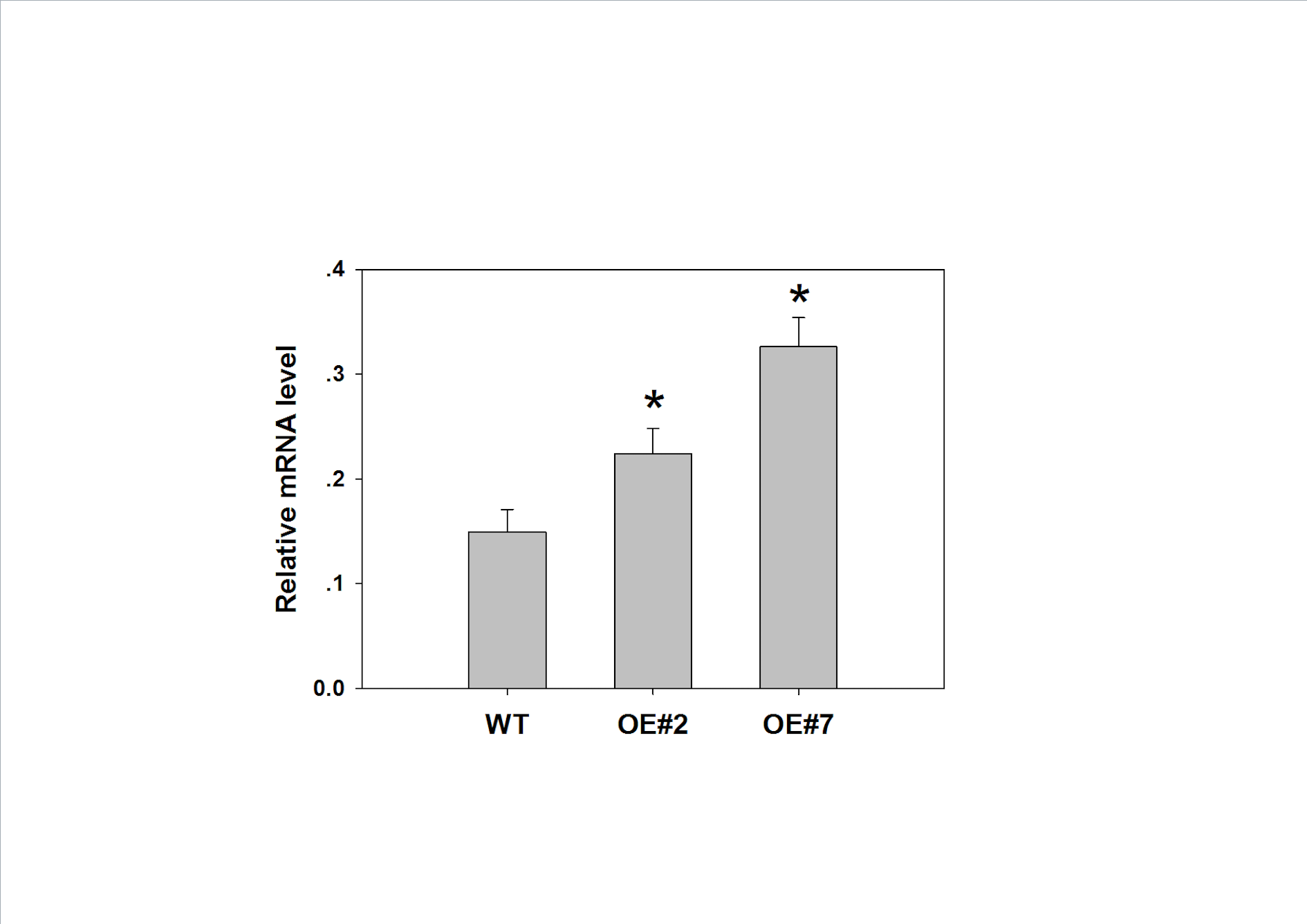

Supplement: Supplementary file 2 — Figure S2 Relative gene expression level of FaHsfA2c in wild‐type tall fescue and transgenic lines. Bars represent ± SD. Asterisk (*) indicates significant difference between each transgenic line and wild type according to Fisher's protected LSD test at a significance level of 0.05. [file PBI-15-237-s002.tif]
