# Supplementary material for: Molecular regulation and physiological functions of a novel FaHsfA2c cloned from tall fescue conferring plant tolerance to heat stress
Source: Plant Biotechnol J. 2016 Sep 23;15(2):237–48. doi: 10.1111/pbi.12609 (PMC5258862; doi:10.1111/pbi.12609)
Supplement: Supplementary file 3 — Table S1 Primers used in the study. [file PBI-15-237-s001.docx]

**Supporting information**

**Table S1** Primers used in the study.

| **Primer Name** | **Forward Primer Sequence (5’-3’)** | **Reverse Primer Sequence (5’-3’)** |
| --- | --- | --- |
| FaHsfA2c | GTGTCATGGACCCGGTGGT | CTTAGGTAAACCATTGTCAACAAAACT |
| RT-FaHsfA2b | CAAATAGCCCGACTCGCACAACTTA | ACACGGTGGAGAATCACTCTGGACA |
| RT-FaHsfA2c | CTGTTGTTTGATTCGGGTGTCCT | CTCAGGTCTCCTTCCCTCCAACTC |
| RT-FaHsfA2d | AGGACCCAACTTCTACGCTATGTG | TTCAGGGGATGAGGAAGGGC |
| FaEF1α | GCGTGACATGAGACAAACGG | AACAGCAGGAAAACTCCAGAC |
| AtActin2 | TGCCAATCTACGAGGGTTTC | TTCTCGATGGAAGAGCTGGT |
| AtApx2 | GGATGGGACTCAATGACAAAGATA | GTCGGTTGGTAGTTGAAGAAGTCC |
| AtHSP18.1 | GGGAAGTTTATGAGAAGGTTTAGGTT | CAAGCCAAGAAAAAAACACAAACT |
| AtHsp22.0 | GCTTGAGAATGGTGTGCTCACTAT | GGTAACTCTCTTCAGACTCAGAAAGTAATA |
| AtHsp25.3 | AAACAGAGGAGGAAGTGGAGTGTCAGA | AATCATCACTGTCTTCCTTCTTCTGCT |
| AtHsp26.5 | CAAAGAGTTATGGTTACTACAACACGA | ACGACACCGTATCTCTTCTACTCAA |
| AtHsp70 | GAAGAGGTGGATTAGAGCGTGTTTAGT | GACACAATACAAAGAAAAGACTCGCTA |
| AtHsp101 | AAAATGCAACCTTTTGGAGCC | TTCATAACCTCTGGACCTTTGAGAC |
| FaApx2 | CTAAACCAAATCCAGCACATCACA | TACTCTGAGGGTTGCTGCTGATTT |
| FaHSP18.1 | TGTCTCCTTCCAGTCCATACGA | CTTCATTTTCCCCTCGCATAGA |
| FaHSP22.0 | CACAGGAAACTCTTCTTACATACGGT | CTCTGAATCTCTGAACTACGAAATGC |
| FaHSP26.5 | GGCAGATAAGCAAGCAAGATAACC | CATTGCTCAAACGAGAATCACAG |
| FaHsp70 | ACTTGCCCAGCAGATTGTTGT | ACCACCATCCCGACCAAGAA |
| FaHsp101 | GGTTCTTGATGATGGGAGGCTA | CCTTCATTGAGTTTCCCACCATT |
